# Role of Polycomb Group Proteins in the DNA Damage Response – A Reassessment

**DOI:** 10.1371/journal.pone.0102968

**Published:** 2014-07-24

**Authors:** Hollie Chandler, Harshil Patel, Richard Palermo, Sharon Brookes, Nik Matthews, Gordon Peters

**Affiliations:** 1 Molecular Oncology Laboratory, Cancer Research UK London Research Institute, London, United Kingdom; 2 Bioinformatics and Biostatistics Service, Cancer Research UK London Research Institute, London, United Kingdom; 3 Advanced Sequencing Facility, Cancer Research UK London Research Institute, London, United Kingdom; The University of Hong Kong, Hong Kong

## Abstract

A growing body of evidence suggests that Polycomb group (PcG) proteins, key regulators of lineage specific gene expression, also participate in the repair of DNA double-strand breaks (DSBs) but evidence for direct recruitment of PcG proteins at specific breaks remains limited. Here we explore the association of Polycomb repressive complex 1 (PRC1) components with DSBs generated by inducible expression of the *Asi*SI restriction enzyme in normal human fibroblasts. Based on immunofluorescent staining, the co-localization of PRC1 proteins with components of the DNA damage response (DDR) in these primary cells is unconvincing. Moreover, using chromatin immunoprecipitation and deep sequencing (ChIP-seq), which detects PRC1 proteins at common sites throughout the genome, we did not find evidence for recruitment of PRC1 components to *Asi*SI-induced DSBs. In contrast, the S2056 phosphorylated form of DNA-PKcs and other DDR proteins were detected at a subset of *Asi*SI sites that are predominantly at the 5′ ends of transcriptionally active genes. Our data question the idea that PcG protein recruitment provides a link between DSB repairs and transcriptional repression.

## Introduction

The Polycomb group (PcG) proteins are important for establishing the patterns of gene expression in different cell types [1]–[3]. They operate within multi-component complexes that associate with and post-translationally modify nucleosomal histones. For example, Polycomb repressive complex 1 (PRC1), which in *Drosophila* comprises equimolar amounts of the Polycomb (Pc), Posterior sex combs (Psc), Polyhomeotic (Ph) and Sex combs extra (Sce) proteins, is credited with the mono-ubiquitination of histone H2A on lysine 119 (H2AK119ub) [1]–[3]. However, as mammalian cells encode several orthologs of each PRC1 component, there can be multiple permutations of the prototypic complex [2]–[4]. In addition, the Psc and Sce components participate in alternative complexes that contain the RYBP/YAF2 proteins rather than Pc and Ph subunits [5]–[7]. As the Psc-Sce dimer is responsible for the ubiquitin ligase activity [8], the extent to which H2A ubiquitination is performed by the canonical or alternate PRC1 complexes has not been formally established [5]–[7], [9], [10].

H2A ubiquitination is also implicated in the repair of DNA double-strand breaks (DSBs) [11], [12]. In mammalian cells, DSBs are mostly repaired by non-homologous end joining (NHEJ) and one of the key events is the recruitment of DNA-dependent protein kinase (DNA-PK) to the DNA ends [13]. DNA-PK comprises a large catalytic subunit (DNA-PKcs), which is a member of the phosphatidylinositol-3-kinase-related-kinase (PIKK) family [14], and two regulatory subunits, Ku70 and Ku80. Following DNA damage, DNA-PKcs becomes auto-phosphorylated on S2056 and is additionally phosphorylated on a cluster of threonine residues by the related PIKK family kinases ATM and ATR [15], [16]. DNA-PK activity is required for re-joining of the DNA ends but not the initial recruitment to the break whereas auto-phosphorylation reduces kinase activity and destabilizes the interaction with DNA ends [15], [17]–[20].

The three PIKK kinases are each capable of phosphorylating the histone 2A variant H2AX on S139 [21], referred to as γH2AX, a modification that occurs within minutes of the DNA damage event and spreads up to a megabase from the site of the break [22]. γH2AX is thought to provide a platform for recruitment and retention of additional DDR proteins, generally in multiple copies, forming a focus that is detectable by immunofluorescence. However, γH2AX is not essential for the initial recognition of the break [23] and is cleared from nucleosomes immediately adjacent to the DSB [24]–[27]. Phosphorylation of H2AX by the PIKK kinases is a prelude for ubiquitination, principally by the RNF8 and RNF168 ubiquitin ligases [28]–[31]. Ubiquitination by RNF168 occurs on residues K13–15 and is therefore distinct from the K119 ubiquitination catalyzed by PRC1 [32], [33]. Nevertheless, a role for PRC1 in the ubiquitination of γH2AX has fostered the idea that it could represent a mechanism linking inhibition of transcription and the repair of DSBs [34].

There are several lines of evidence suggesting that PRC1 proteins are involved in the repair of DSBs. First, a number of PRC1 proteins have been found to co-purify with γH2AX and other DDR proteins and their association is enhanced by DNA damage [35]–[37]. Second, cells lacking specific PRC1 proteins are reported to be more sensitive to DNA damage [36]–[41]. The bulk of the evidence, however, relates to immunofluorescence experiments in which PRC1 proteins were found to co-localize with DDR proteins at sites of DNA damage induced by ionizing radiation, genotoxic drugs or laser micro-irradiation [35], [36], [39], [41]. A limitation of this approach is that the sites of DNA damage are random and therefore differ from cell to cell, precluding attempts to demonstrate co-association at specific DSBs. The interpretation is further complicated by the fact that many of the studies were conducted in transformed cell lines in which PRC1 proteins concentrate in large nuclear bodies associated with peri-centromeric heterochromatin [42]–[49]. Although these features are commonly referred to as Polycomb bodies, it is not clear whether they are functionally equivalent to the smaller and more dispersed Pc bodies observed in non-transformed cells and in *Drosophila* embryos [45], [46], [50], [51] reviewed in [52].

Using a panel of antibodies that support chromatin immunoprecipitation (ChIP) of endogenous PRC1 proteins in normal human fibroblasts (HFs), we previously established that multiple PRC1 components co-localize at common sites in the genome [53]. Our ability to perform sequential ChIP with antibodies against different Pc, Ph and Sce orthologs implied that multiple permutations of the canonical PRC1 complex are associated with the same DNA, suggesting that they act collectively in what we suspect are the mammalian equivalents of the Pc bodies described in *Drosophila*. Here we investigated whether multiple PRC1 complexes also congregate at specific DSBs generated by conditional expression of the *Asi*SI restriction enzyme in primary HFs. Contrary to expectations, we found no evidence that PRC1 proteins are stably associated with persistent DSBs.

## Materials and Methods

### Cell culture and retroviral infection

The BF strain of human breast fibroblasts [54] and the Hs68 strain of human foreskin fibroblasts (ATCC: CRL 1635) were propagated as previously described [55]. U2OS cells expressing *Asi*SI:ER and a pBABE-based retroviral vector encoding the HA-tagged *Asi*SI:ER fusion protein were generously provided by Dr Gaëlle Legube [26]. To generate infectious viral particles, the vector was transfected into 293 T cells expressing the structural components for amphotropic retroviruses. Medium was harvested after 24–36 h, filtered through a 0.45 µm Millex-HV filter (Millipore) and used directly. The recipient cells were passaged (1∶4) into 100 mm culture dishes 24 h prior to infection. The medium was replaced with 10 ml of the filtered viral supernatant. After 48 h, the cells were washed and placed in selection medium containing 0.75 µg/ml puromycin (Invitrogen).

To activate the *Asi*SI:ER fusion protein, cells were grown to near confluence and incubated in medium containing 4-hyrodoxy tamoxifen (OHT) at a final concentration of 300 nM. Control cells received an equivalent amount of methanol, which was used as a solvent for the OHT. For most experiments, cells were fixed after 4 h and analyzed by either immunofluorescence or chromatin immunoprecipitation (ChIP).

### Antibodies

The antibodies used for different applications in this study are listed in Table S1.

### Immunostaining and microscopy

Approximately 10^3^ cells were seeded on coverslips in 12-well culture plates and after 24 h they were washed once in PBSA for 1 min and fixed in 3.7% formaldehyde in PBSA for 15 min at room temperature. Cells were then subjected to 4×20 sec washes in PBSA, permeabilized in 0.1% Triton X-100 in PBSA for 15 min at room temperature and washed again. Four drops of Image-iT FX Signal Enhancer (Molecular Probes) were added to each well and incubated for 30 min at room temperature in a humid atmosphere. After incubation, cells were washed and blocked in 3% BSA in PBSA for 1 h. Cells were then incubated with primary antibody (previously diluted in 3% BSA in PBSA) overnight at 4°C or for 1 h at room temperature. See Table S1 for details of the antibodies used. After washing, the relevant fluorescein-coupled (Alexa Fluor 488 or 555) secondary antibody, diluted in 3% BSA in PBSA, was applied and incubated for 30–60 min at room temperature in the dark. Cells were washed and coverslips were mounted onto glass microscopy slides using ProLong Gold Antifade reagent with DAPI (Invitrogen).

Images were acquired using a 40x/1.3 DIC Plan Apochromat lens under oil immersion and a Zeiss LSM *invert* 710 microscope using sequential scanning. Zen 2009 software (Zeiss) was used. DAPI was excited using a 405 nm laser line, the Alexa Fluor 488 with a 488 nm laser line and the Alexa Fluor 555 with a 561 nm laser line. Spatial sampling was 0.04 µm per pixel in the X/Y plane and between 0.2 µm and 0.3 µm per pixel in the Z plane. The pinhole aperture was set to 1 airy unit. For each antibody pair, Z-stacked images of three representative nuclei were collected and the images were deconvoluted using Huygens Essential software (SVI). Co-localization analyses were performed using Imaris 7.6 software (Bitplan) and automatically selected intensity thresholds in the region of interest, (set by the DAPI channel) to generate Pearson’s correlation coefficient (PCCs) values.

### Chromatin immunoprecipitation assays and DNA sequencing

ChIP assays were performed as described [53]. After sonication to obtain chromatin fragments of between 200 and 1000 base pairs (bp), solubilized chromatin was diluted to 1 µg/µl and incubated with the appropriate antibody at 4°C overnight. The antibodies are listed in Table S1 and a species-matched irrelevant antibody was used as control. After reversal of the crosslinks, the immunoprecipitated DNA was quantified by qPCR with the primer sets described in Table S2.

To generate sufficient quantities of DNA for sequence analyses, parallel ChIPs were performed using approximately 5 µg of antibody with 500 µg chromatin. The recovered material was pooled and concentrated to a minimum of 0.2 µg/µl. DNA samples were end-repaired, poly-A tailed and Illumina single-end adapters were ligated following the standard Illumina protocol with minor adjustments. Agencourt AMPure XP beads at 0.8x ratio were used to size select out adapter dimers after adapter ligation. The Illumina kit Phusion enzyme was replaced by Kapa HiFi HotStart ready mix. Post PCR, AMPure XP beads were used at a 1∶1 ratio to maintain size integrity and to allow use of the Invitrogen SizeSelect E-gel system. Samples were finally purified with QAIquick gel extraction kit and quality controlled on the DNA 1000 BioAnalyser 2100 chip before clustering and subsequent 50–51 bp single end sequencing on the Illumina HiSeq 2500.

### Bioinformatics

Fastq files containing the sequenced reads were merged for technical replicates. Where required, reads were trimmed to 50 bp and those with greater than two Ns were removed prior to alignment. Alignments were performed using novalign (version 2.07.14: http://novocraft.com) with default parameters and subsequently filtered to allow for a single mismatch per read. Duplicate reads were removed using the Picard MarkDuplicates programme (picard-tools package version 1.81; http://picard.sourceforge.net) with default parameters. Peak calling was performed using MACS (version 1.4.Orc2; ref [56]) and downstream annotation to gene intervals and *Asi*SI sites was carried out using the “annotatePeaks.pI” programme (HOMER software suite version 4.1; ref [57]) and BEDTools package (version 2.17.0; ref [58]), respectively.

### Quantitative real-time PCR (qPCR)

All reactions were performed in triplicate in 96-well plates (Applied Biosystems) with the following combination of reagents: 2 µl DNA, 10 µl H_2_O, 0.5 µl primer mix (10 µM each of the forward and reverse primers) and 12.5 µl Express qPCR Supermix with premixed ROX (Invitrogen). The primer sets are listed in Table S2. After sealing the wells, PCR cycling was carried out in a 7500 FAST Real-Time PCR machine (Applied Biosystems). Data were analyzed using SDS software (Applied Biosystems) and exported to Excel.

## Results

### Inducing specific DSBs in normal human fibroblasts

**Figure 1:**
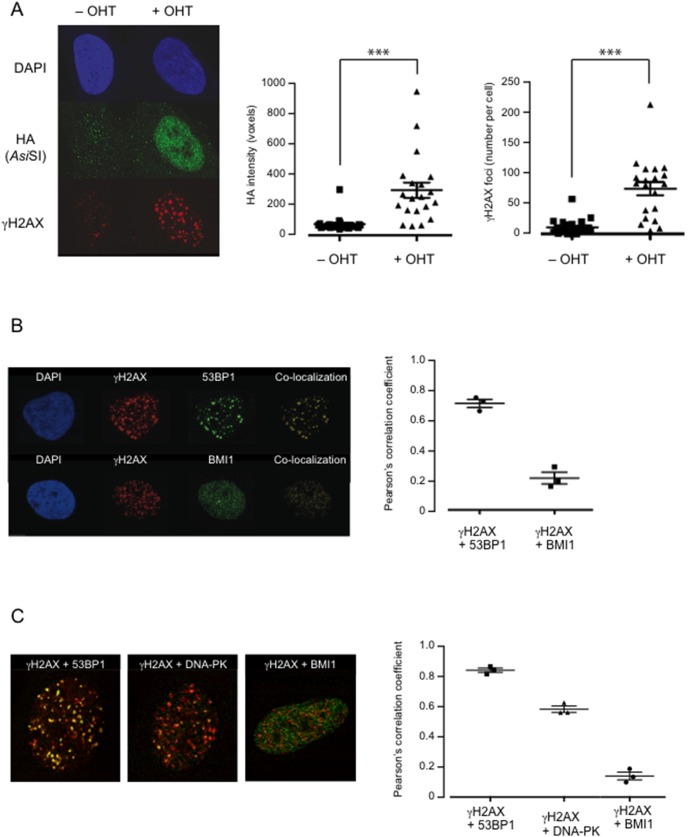
Visualizing *Asi*SI-induced DNA damage foci in HFs by indirect immunofluorescence. **A.** *Asi*SI:ER-transduced Hs68 cells were treated for 4 h with or without OHT and co-stained with DAPI and antibodies against either the HA-tag on the fusion protein (green) or γH2AX (red). Right panels show quantification of the HA intensity (voxels) and number of γH2AX foci in 20 representative nuclei, +/− OHT. Error bars represent the standard deviation and *** signifies a P value<0.001 in a student’s t-test. **B.** Similar analyses comparing the staining for γH2AX (red) and either 53BP1 or BMI1 (green). Images were deconvoluted using Huygens Essential software and the Imaris program was used to generate a co-localization channel (yellow). Right panel shows Pearson’s correlation coefficient values for the indicated pairs of markers in three representative nuclei. **C.** An equivalent experiment comparing staining for γH2AX (red) and either 53BP1, pDNA-PKcs, or BMI1 (green).

To avoid potential anomalies associated with tumor cell lines, we sought a system for inducing specific DSBs in normal, non-transformed human cells. To this end, we exploited the previously described fusion protein between the *Asi*SI restriction enzyme and a modified hormone-binding domain from the estrogen receptor [26], [27] and used a retroviral vector to express the epitope-tagged protein in two strains of primary HFs (BF and Hs68). Following drug selection, the cell populations were treated with OHT or solvent control for different times and the effects were analyzed by indirect immunofluorescence. Exposure to OHT resulted in nuclear accumulation of the *Asi*SI fusion protein, as detected with an antibody against the HA-tag (Figure 1A). This was accompanied by a significant increase in the number of DNA damage foci, visualized with an antibody against γH2AX (Figure 1A). In line with previous reports, the signal intensity reached a plateau after approximately 4 h (not shown). Although small numbers of γH2AX foci were observed in uninfected cells and in the untreated controls, the numbers did not increase upon continued passaging, suggesting that the system was not inherently leaky. However, the majority of experiments were conducted in freshly infected cell populations. Because of their variable size and intensity, we did not attempt to estimate the total number of visible foci but it appeared to be substantially lower than the number of predicted *Asi*SI recognition sites. There are 1219 exact matches of the 8 bp *Asi*SI recognition site (GCGATCGC) in the human genome but as the sequence is subject to CpG methylation, only a subset of sites are likely to have been cleaved [26]. These preliminary observations in HFs paralleled the effects observed in a clonal population of U2OS cells expressing the *Asi*SI:ER fusion protein (Figure S1 and [26]).

### Location of PRC1 and DDR proteins following induction of *Asi*SI

**Figure 2:**
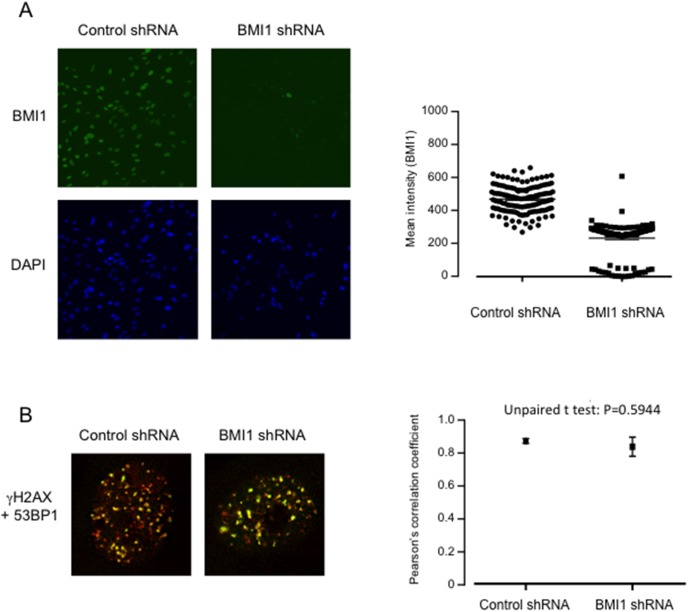
Visualizing *Asi*SI-induced DNA damage foci following BMI1 depletion. **A.** *Asi*SI:ER-transduced Hs68 cells were infected with a lentiviral vector expressing an shRNA against BMI1, or a random control shRNA, and stained for BMI1 (green) or DAPI. The right panel shows quantification of the BMI1 staining intensity (voxels) in 20 representative nuclei. **B.** The cells were treated with OHT for 4 h, to activate *Asi*SI, and co-stained for γH2AX (red) and 53BP1 (green). The right panel shows Pearson’s correlation coefficient values for co-localization of γH2AX and 53BP1 with and without BMI1 depletion.

Earlier studies reported that, in normal HFs, PRC1 proteins are found in multiple speckles throughout the nucleus [45], [46], [52]. In line with these reports, staining of the *Asi*SI:ER-transduced HFs with a widely used monoclonal antibody against the Psc ortholog BMI1 revealed a diffuse, granular distribution in the nucleus (Figure 1B). The provenance of the antibody was confirmed by loss of the fluorescence signal following shRNA-mediated knockdown of BMI1 (see Figure 2A). Note that the staining pattern in HFs is quite different from the situation in the U2OS cell line where PRC1 proteins are concentrated in large bodies (Figure S1B and [42]–[49]).

To investigate whether BMI1 co-localized with γH2AX and other DDR proteins at *Asi*SI-induced DSBs, cells treated with OHT were co-stained with different pairs of mouse and rabbit antibodies and the signals were detected using either Alexa 488- or Alexa 555-conjugated secondary antibodies (Figure 1B and 1C). In independent experiments, there was very clear evidence for co-localization of γH2AX and 53BP1, with a high Pearson’s correlation coefficient (PCC ∼0.8), and also between γH2AX and the S2056-phophorylated form of DNA-PKcs (PCC 0.6). In contrast, the correlation between γH2AX and BMI1 staining was weak (PCC ≤0.2) and according to a range of statistical tools would not be considered as strong evidence for co-localization [59]. We have thus far been unable to visualize other endogenous PRC1 proteins in HFs with a similar degree of confidence.

For comparison, we conducted parallel analyses in U2OS cells expressing the *Asi*SI:ER fusion protein. Whereas γH2AX and 53BP1 clearly co-localized, there was effectively no correlation between γH2AX and BMI1 staining (Figure S1B). A similar result was obtained for γH2AX and RING2 (Figure S1B). In the U2OS cell background, we were able to detect endogenous RING1 and MEL18, presumably because of their concentration in nuclear bodies. This allowed us to confirm the co-localization of the two Psc orthologs (BMI1 and MEL18) and both Sce orthologs (RING1 and RING2), as previously reported [42]–[49]. Taken together, our data provided little support for the idea that PRC1 proteins are quantitatively recruited to *Asi*SI-induced DSBs.

### Effect of BMI1 depletion on DDR recruitment at *Asi*SI-induced DSBs

Several studies have suggested that genetic or shRNA-mediated depletion of individual PRC1 proteins impairs the DDR and renders cells more sensitive to DNA damage [36]–[41]. To investigate whether this holds true in HFs, we used a previously validated shRNA [60] to knock down the levels of BMI1 in Hs68 cells expressing *Asi*SI:ER (Figure 2A). The cells were then treated with OHT for 4 h and co-stained for γH2AX and 53BP1. The induction of DNA damage foci and co-localization of the two proteins appeared to be unaffected by BMI1 depletion (Figure 2B). As HFs express an extensive repertoire of PRC1 components, it is possible that other proteins compensate for the loss of BMI1. However, knockdown of BMI1 resulted in de-repression of p16^INK4a^ and a senescence-like growth arrest as previously described [60], effectively precluding attempts to perform survival assays. 

### Chromatin association of DDR and PRC1 proteins at *Asi*SI-induced DSBs

**Figure 3:**
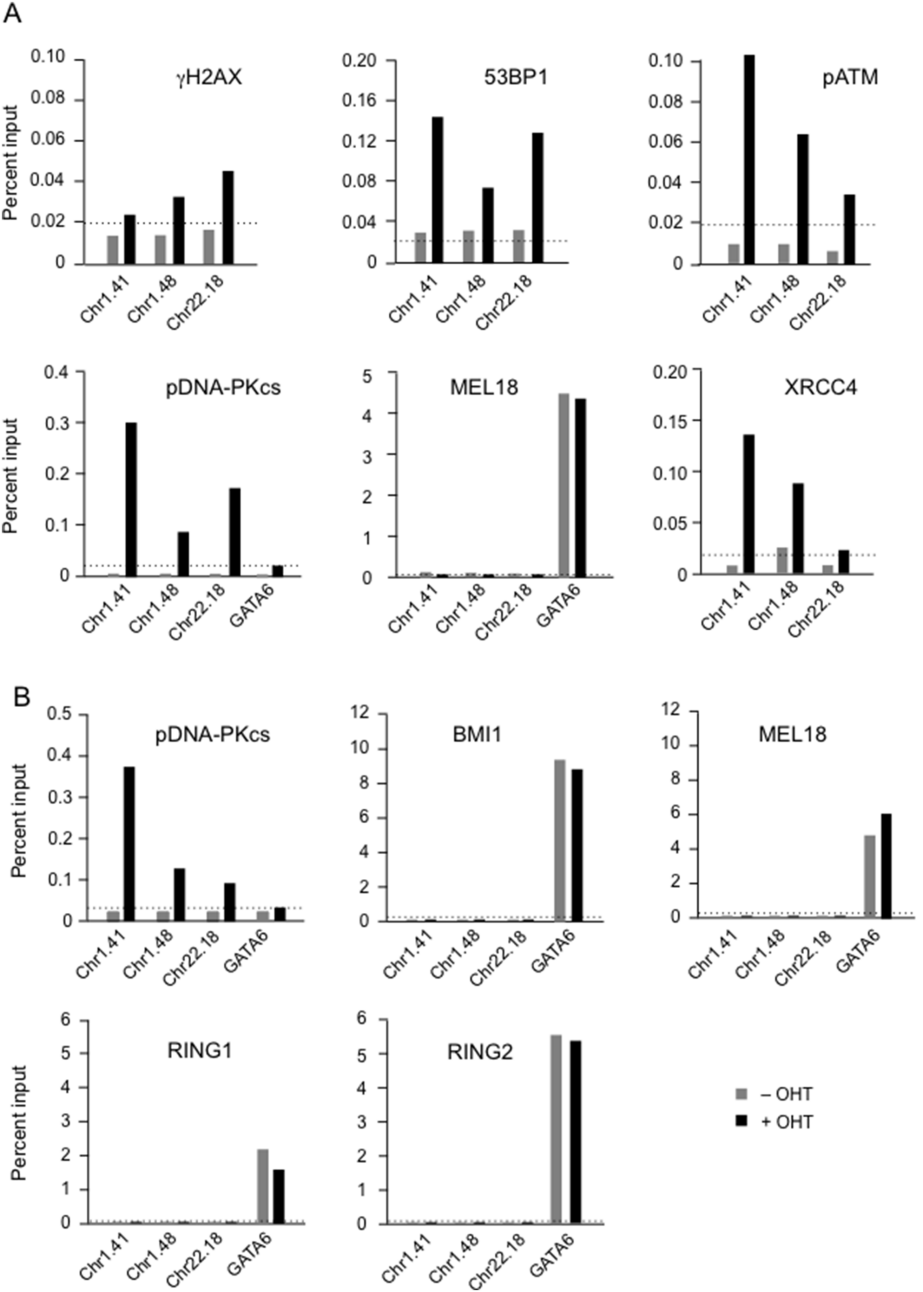
Detection of DDR proteins at representative *Asi*SI sites by chromatin immunoprecipitation. **A.** Chromatin immunoprecipitation (ChIP) assays were performed in *Asi*SI:ER-transduced Hs68 cells, before and after addition of OHT, using antibodies against γH2AX, 53BP1, pATM, XRCC4, pDNA-PKcs and MEL18 as indicated. Enrichment was assessed by real-time qPCR using primers adjacent to the Chr1.41, Chr1.48 and Chr22.18 *Asi*SI sites or from the PcG target gene *GATA6* (see Table S2). **B.** Equivalent ChIP assays performed in the BF cell background with antibodies against pDNA-PKcs, BMI1, MEL18, RING1 and RING2. The data are from single representative experiments showing the average of triplicate PCR reactions plotted as a percentage of input. An irrelevant IgG control was included in each experiment and the dotted line shows the mean enrichment in OHT treated cells.

The reason for generating the *Asi*SI:ER-expressing HFs was to enable us to use ChIP-based approaches to investigate whether DDR and PRC1 proteins are enriched in chromatin adjacent to specific DSBs. As an initial test, we focused on a number of *Asi*SI sites on chromosomes 1 and 22 at which γH2AX recruitment had been observed in U2OS cells [26]. We conducted ChIP with antibodies against a variety of DDR components using the published primer sets (listed in Table S2). As illustrated in Figure 3, we observed increased enrichment of γH2AX, 53BP1, pDNA-PKcs, pATM and XRCC4 at the Chr1.41, Chr1.48 and Chr22.18 sites following OHT-induced activation of *Asi*SI. Analogous results were obtained in different fibroblast strains but there was considerable variation in the signals obtained with different antibodies and primer sets. The readout from the ChIP assay could depend on many factors, such as the efficacy of the antibody, the accessibility of the protein in the complex and its location relative to the DSB but, collectively, the results implied that these three *Asi*SI sites were susceptible to enzymatic cleavage in the OHT-treated HFs, as in U2OS cells.

To assess whether PcG proteins were also recruited to these sites, the same chromatin was interrogated with ChIP-validated antibodies against a number of PRC1 proteins. As we recently reported, the PRC1 components expressed in HFs have very similar if not identical binding profiles throughout the genome, suggesting that they act collectively [53]. If they also act collectively at DSBs, then we would have expected to see a ChIP signal at *Asi*SI sites with each of the antibodies. In the event, none of the PRC1 antibodies tested showed detectable enrichment with primers for the three *AsiS*I sites, under conditions that revealed robust binding at known PRC1 target genes, such as *GATA6* (Figure 3). Analogous results were obtained in both strains of HF and with antibodies against BMI1, MEL18, RING1, RING2 and CBX6 (Figure 3 and additional data not shown). Conversely, the DDR proteins did not show significant enrichment at *GATA6*. Note that the ChIP signals for the PRC1 components were generally an order of magnitude higher than those achieved with the DDR proteins.

### Genome-wide profiling of PRC1 and DDR proteins following *Asi*SI-induced DNA damage

The PCR-based analyses only sampled limited regions of DNA, dependent on primer design, and could in principle have missed the presence of PRC1 components in the vicinity of DSBs. For a more unbiased view, we subjected the precipitated chromatin to deep sequencing (ChIP-seq). As the positive control for recruitment of DDR proteins to *Asi*SI sites, we used the antibody against pDNA-PKcs, based on the consistency and degree of enrichment achieved with this reagent in the pilot studies. Applying the same criteria, MEL18 was chosen as a representative of PRC1. ChIP-seq was performed on equivalent numbers of *Asi*SI:ER-expressing Hs68 cells that were treated with or without OHT for 4 h. Sequencing was performed on the Illumina HiSeq 2500 platform and typically yielded 35 million 50 bp reads that could be aligned with the hg19 release of the human genome. The raw and processed data have been deposited under GEO accession number GSE55605.

**Figure 4:**
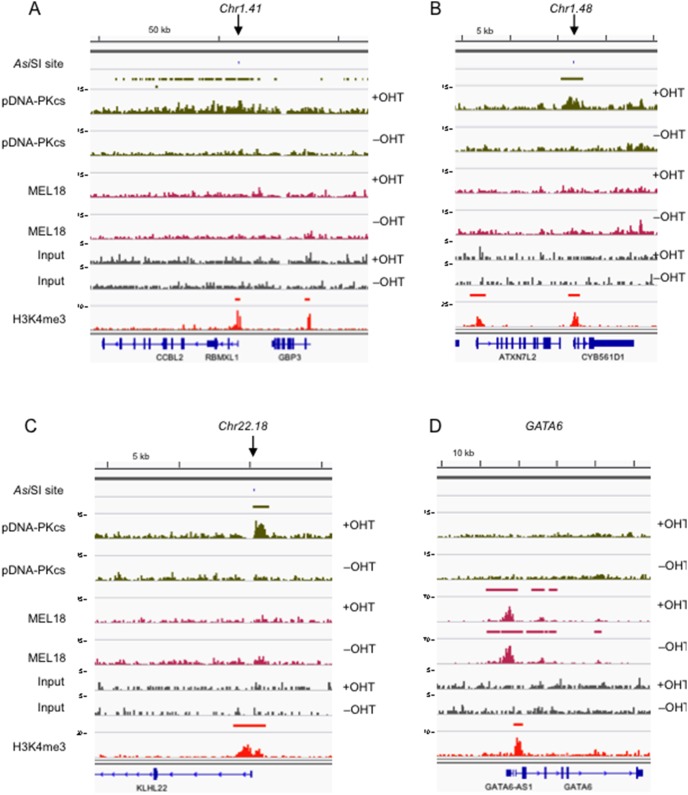
Examples of ChIP-seq data at representative *Asi*SI sites and PcG target loci. The panels show DNA sequence tag densities following ChIP-seq with the pDNA-PKcs and MEL18 antibodies in *Asi*SI:ER-transduced Hs68 cells, before and after addition of OHT, as indicated. Input refers to parallel analyses of the chromatin before immunoprecipitation. The maximum coverage for each track is shown on the left of the IGV (Integrative Genomics Viewer www.broadinstitute.org/igv/) screen shot and a size bar is included above. The locations of the Chr1.41, Chr1.48 and Chr22.18 *Asi*SI sites are identified by a downward arrow (panels **A**, **B** and **C** respectively) and the genomic organization of adjacent RefSeq loci is shown below. *GATA6*, a known PRC1 target gene, is included as a positive control (panel **D**). The H3K4me3 track refers to genome-wide enrichment of H3K4me3 in the Hs68 strain of HFs from a previously deposited dataset (GEO accession number 40740).

Focusing initially on the representative *Asi*SI sites analyzed above, standard peak calling algorithms, such as MACS [56], identified significant enrichment for pDNA-PKcs at the Chr1.41, Chr1.48 and Chr22.18 sites, specifically in the cells treated with OHT (Figure 4). In contrast, there was no apparent enrichment for MEL18 at these sites, irrespective of OHT treatment, although robust binding was detected at *GATA6*, in line with our previous observations (Figure 4 and [53]).

**Figure 5:**
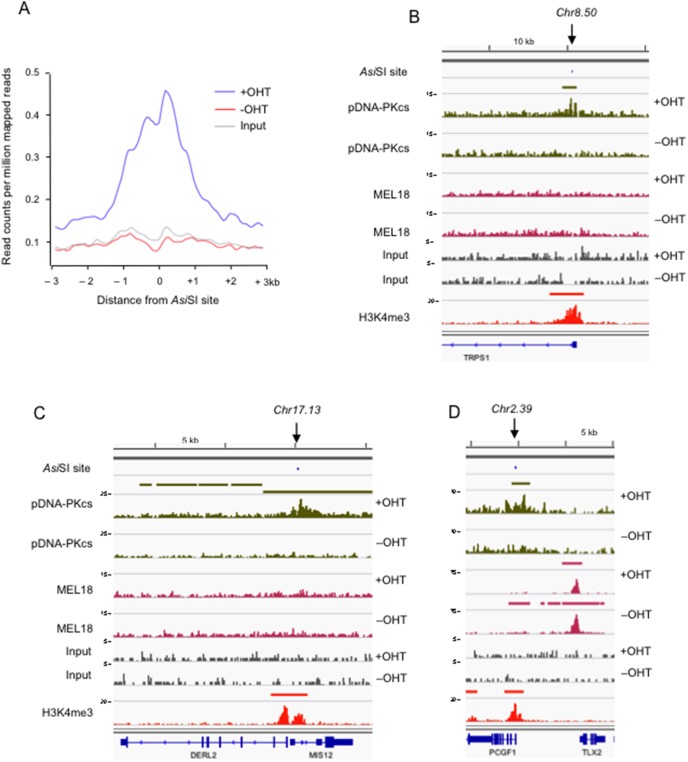
ChIP-seq profiles of pDNA-PKcs and PRC1 binding at *Asi*SI sites. **A**. The graph plots the read-count per million mapped reads for pDNA-PKcs binding in OHT treated (blue) and untreated (red) cells with input in grey at the 108 *Asi*SI sites listed in Table S3, compiled using ngsplot (https://code.google.com/p/ngsplot/). **B, C** and **D** show examples of the DNA sequence tag densities following ChIP-seq with the pDNA-PKcs and MEL18 antibodies in *Asi*SI:ER-transduced Hs68 cells, before and after addition of OHT, as indicated. Input refers to parallel analyses of the chromatin before immunoprecipitation. The maximum coverage for each track is shown on the left of the screen shot and a size bar is included above. The locations of the Chr8.50, Chr17.13 and Chr2.39 *Asi*SI sites are identified by a downward arrow and the genomic organization of adjacent RefSeq loci is shown below. The H3K4me3 track refers to genome-wide enrichment of H3K4me3 in the Hs68 strain of HFs from a previously deposited dataset (GEO accession number 40740).

Knowing the locations of predicted *Asi*SI sites in the genome, we then investigated whether pDNA-PKcs was recruited to any or all of these sites in the cells treated with OHT. Setting a limit of +/− 1 kb from the break, pDNA-PKcs binding was detected at 111 (9.1%) of the *Asi*SI sites (summarized in Table S3). Increasing the range only marginally increased this percentage, suggesting that pDNA-PKcs binds predominantly at or adjacent to the DSB. Indeed, meta-analyses of the binding profiles at *Asi*SI sites confirmed the tight association with the DSB (Figure 5A) and at 92 locations, the pDNA-PKcs peak overlapped an *Asi*SI site, or in some cases a cluster of sites (see examples in Figure S2A). However, there was considerable variability in the shape of the peaks and their positions relative to the DSB, ranging from single well-defined peaks of around 0.5 to 2.0 kb (for example Chr1.48 and Chr22.18 in Figure 4 and Chr8.50 in Figure 5B), to broader regions of enrichment (for example Chr17.13 in Figure 5B), to blocks of MACS peaks that extended for considerable distances (≥100 kb) on either side of the DSB (for example, Chr1.41 in Figure 4, and Chr18.11 and Chr2.68 in Figures S2B and S2C). It is not clear whether the latter situation reflects spreading of the DDR focus, as described for γH2AX [26], [27] and a more comprehensive analysis would be required to address this issue and the reasons for the varied patterns. In the only comparable study that we are aware of, ChIP-seq defined 105 γH2AX domains in *Asi*SI-expressing T98G cells [27].

In striking contrast to the pDNA-PKcs distribution, MEL18 was detected at multiple sites throughout the genome with a profile that was virtually identical to the other PRC1 proteins that we analyzed previously [53]. The *HOXD* cluster and other examples are shown in Figure S3. Importantly, there was no evidence that MEL18 was recruited to *Asi*SI sites or that its distribution was altered in the cells treated with OHT. To illustrate this point, Figure 5D shows a situation in which an *Asi*SI site (Chr2.39), in the promoter region of the *PCGF1* gene, is juxtaposed to a known PRC1 target gene, *TLX2*. Whereas pDNA-PKcs was detected at *PCGF1,* specifically in the OHT-treated cells, there was no corresponding OHT-dependent enrichment for MEL18. In contrast, MEL18 showed very robust enrichment at *TLX2* in both control and treated cells, with the same characteristic binding pattern as other PRC1 proteins (Figure 5D and [53]). Of the 1219 predicted *Asi*SI sites, 41 were located within known PRC1 binding domains. However, only 7 of the OHT-dependent pDNA-PKcs peaks overlapped with a MEL18 peak and the presence of MEL18 at these sites was unaffected by addition of OHT (e.g. Chr12.11/*ABCC9*, Chr9.38/*NR6A1* and Chr20.27/*SLC32A1* in Figure S4).

### Association of pDNA-PKcs binding with transcription start sites

In compiling the list of *Asi*SI sites that were bound by pDNA-PKcs (Table S3), we noted that the majority of the sites occurred at or near the 5′ end of an annotated gene. This impression was reinforced by the profile of H3K4me3, a mark generally associated with transcriptionally active promoters, that we had generated in our previous study [53]. Importantly, at 95 (88%) of the 108 locations where we observed a pDNA-PKcs peak within 1 kb of an *Asi*SI site(s), there was also an H3K4me3 peak (see Chr1.41, Chr1.48 and Chr22.18 in Figure 4, and Chr8.50, Chr17.13 and Chr2.39 in Figure 5, and additional examples in Figures S2 and S4). A possible interpretation is that the chromatin remodelling associated with active transcription determines whether the *Asi*SI site is accessible to cleavage by the enzyme. However, the correlation is not perfect as there were examples of *Asi*SI sites in H3K4me3-positive promoter regions that were not bound by pDNA-PKcs as well as peaks of pDNA-PKcs binding that did not coincide with active promoters (for example, Chr20.27/*SLC32A1* in Figure S4C).

**Figure 6:**
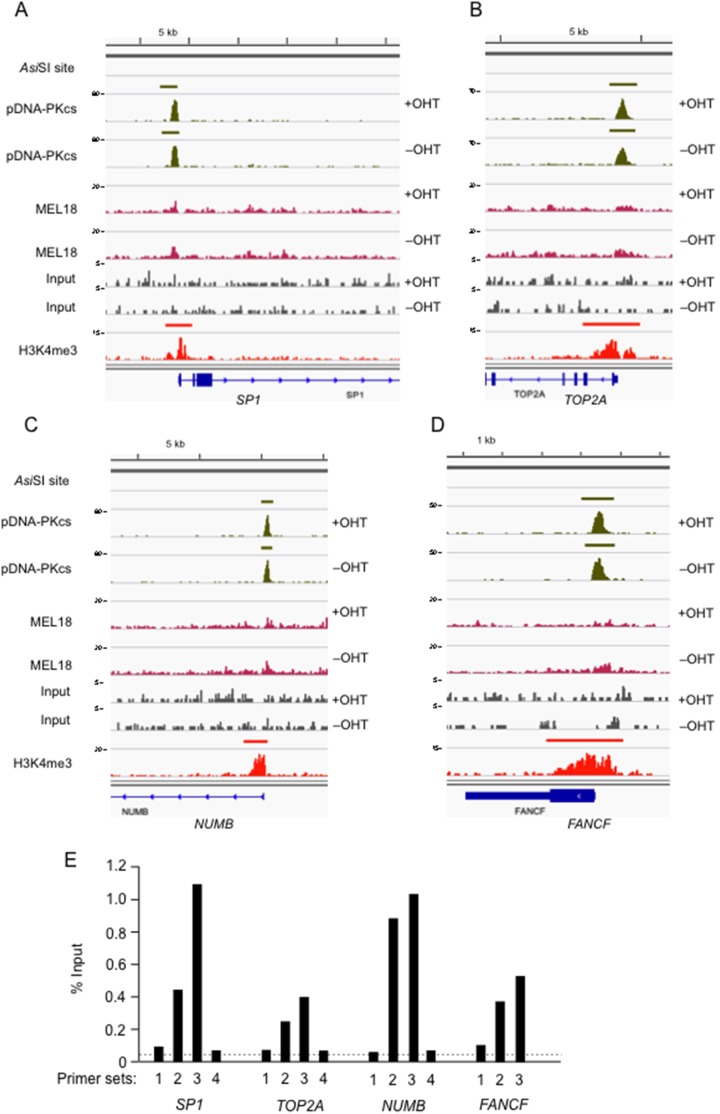
Detection of pDNA-PKcs at promoters irrespective of DNA damage. Panels **A–D** show DNA sequence tag densities following ChIP-seq with the pDNA-PKcs and MEL18 antibodies in *Asi*SI:ER-transduced Hs68 cells, before and after addition of OHT, as indicated. Input refers to parallel analyses of the chromatin before immunoprecipitation. The maximum coverage for each track is shown on the left of the screen shot and a size bar is included above. The peaks do not correspond to *Asi*SI sites and are unaffected by OHT-induction of *Asi*SI. The genomic organization of adjacent RefSeq loci is shown below. The H3K4me3 track refers to genome-wide enrichment of H3K4me3 in the Hs68 strain of HFs from a previously deposited dataset (GEO accession number 40740). **E.** Confirmation of pDNA-PKcs binding at the indicated loci in normal Hs68 cells. The precipitated DNA was subjected to qPCR with panels of primers that spanned the TSS of the *SP1, TOP2A*, *NUMB* and *FANCF* loci (see Table S2). The dotted line shows the mean enrichment observed with an irrelevant IgG control antibody.

We also noted that there was a substantial number of prominent pDNA-PKcs peaks that were present in both the OHT-treated and untreated samples (Figure 6A–D). Representative examples were validated by qPCR with a series of primers that reflected the profiles of the peak (Figure 6E). Of the 785 peaks in this category, only 9 were within +/− 1 kb of an *Asi*SI site. The peaks were well-defined and 610 (78%) of them were associated with the promoter regions of annotated genes. Again, the vast majority (94%) of these genes were deemed to be active as judged by H3K4me3 profiles. Interestingly, several loci showed distinct pairs of pDNA-PKcs and H3K4me3 peaks associated with different transcription start sites (see examples in Figure S5). However, we have thus far been unable to detect other DDR-associated proteins at these locations and the role, if any, of DNA-PKcs at these promoters remains uncertain.

**Figure 7:**
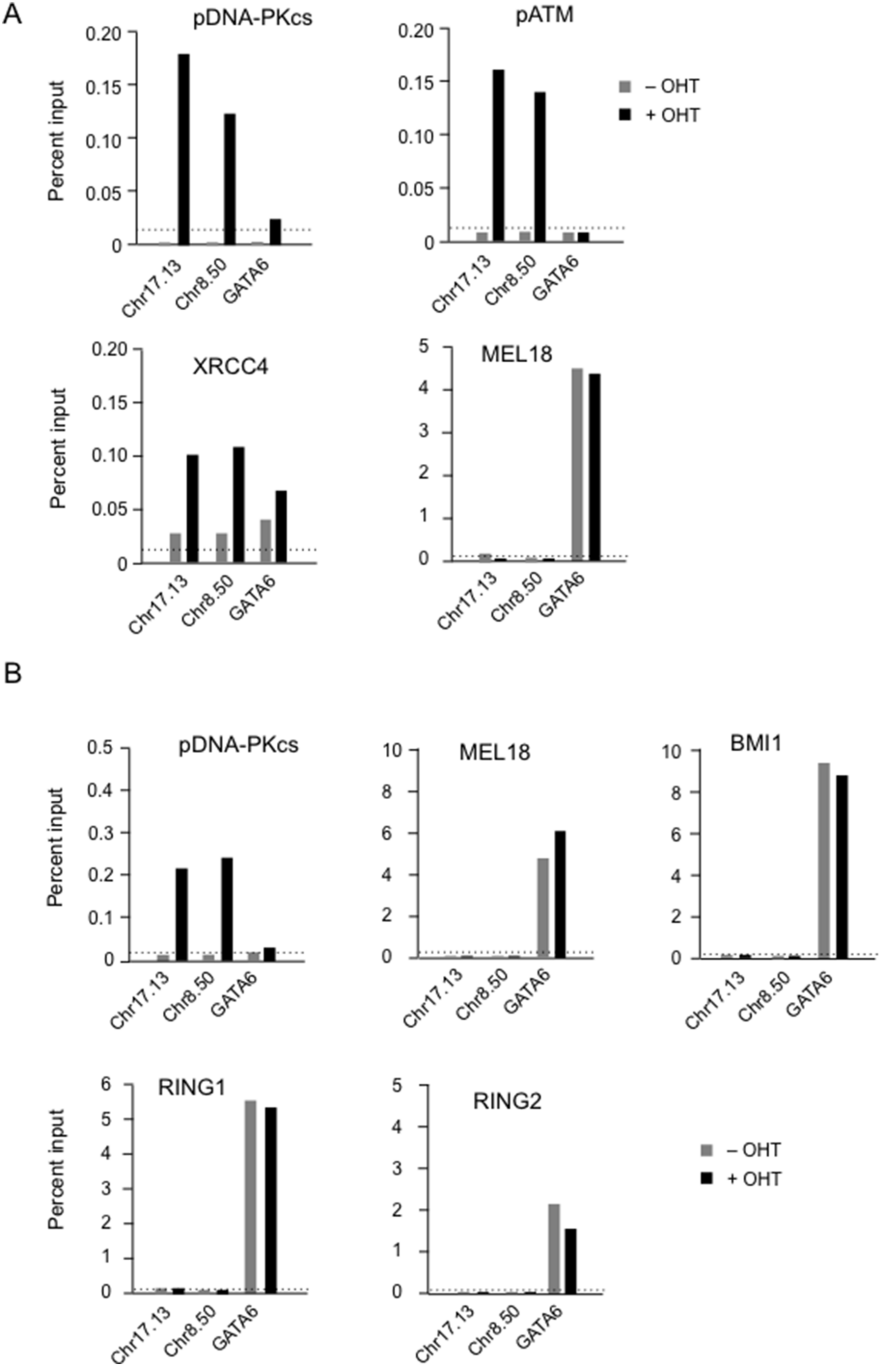
ChIP-PCR validation of DDR protein recruitment at selected *Asi*SI sites. **A.** ChIP assays were performed in *Asi*SI:ER-transduced Hs68 cells, before and after addition of OHT, using antibodies against pDNA-PKcs, pATM, XRCC4 and MEL18 as indicated. Enrichment was assessed by real-time qPCR using primers adjacent to the Chr17.13 and Chr8.50 *Asi*SI sites or from the PcG target gene *GATA6* (see Table S2). **B.** Equivalent ChIP assays performed in the BF cell background with antibodies against pDNA-PKcs, BMI1, MEL18, RING1 and RING2. The data are from single representative experiments showing the average of triplicate PCR reactions plotted as a percentage of input. An irrelevant IgG control was included in each experiment and the dotted line shows the mean enrichment in OHT-treated cells.

In this regard, the OHT-independent peaks were quite distinct from the hundred or more pDNA-PKcs peaks that were associated with *Asi*SI sites following induction of the enzyme with OHT. As for the previously described examples shown in Figure 3, we confirmed that a number of the novel sites represent bona fide DSBs, based on the recruitment of DDR proteins (Figure 7). Thus, qPCR-based assays detected pDNA-PKcs, pATM and XRCC4 at the Chr8.50 and Chr17.13 sites, but only in OHT-treated cells. However, there was no evidence for binding by BMI1, MEL18, RING1 or RING2 at the *Asi*SI sites under conditions in which they were readily detected at PRC1 target loci. These findings reinforced our conclusion that PRC1 proteins are not detectable at DSBs generated by the *Asi*SI restriction enzyme.

## Discussion

The concept that PcG proteins are involved in the DDR has an appealing logic as well as a considerable body of experimental support. However, inconsistencies in the published evidence and the data we describe here call for a more cautious interpretation. By conducting the analyses in primary HFs, we have avoided concerns that PRC1 localization is distorted in transformed cell lines and, by using *Asi*SI to generate DSBs, we have been able to assess the recruitment of proteins at multiple, defined sites. Moreover, the breaks reflect simple hydrolysis events that generate 3′-hydroxyl and 5′-phosphate ends, without the potential for collateral damage associated with ionizing radiation. Finally, by exploiting ChIP-seq, we have obtained a relatively unbiased genome-wide impression, unlike systems that introduce breaks at single sites or in atypical regions of chromatin [24], [25], [61].

With regards to the DDR, the cell system behaved as anticipated. Addition of OHT resulted in increased numbers of γH2AX foci at which we could detect the co-localization of other DDR proteins, including 53BP1 and the S2056 phosphorylated form of DNA-PKcs, consistent with the role of this protein in NHEJ. In addition, ChIP analyses confirmed the recruitment of several DDR proteins at representative DSBs, in line with previous reports [26], [27], and the genome wide profiling of pDNA-PKcs by ChIP-seq revealed significant binding adjacent to a subset of *Asi*SI sites.

There were, however, two unexpected findings that warrant further investigation. First, the *Asi*SI sites at which we detected pDNA-PKcs were predominantly associated with the 5′ ends of transcriptionally active genes, suggesting a link between nucleosome density and accessibility to the restriction enzyme. Second, there was a substantial number of prominent, well-defined pDNA-PKcs peaks that were not associated with *Asi*SI sites and were of equivalent magnitude in the control and OHT-treated cells. Most of these peaks were again at or near the TSS of an H3K4me3-marked gene. However, as we have been unable to confirm the presence of other DDR proteins at these locations, their relevance remains unclear at this point. Importantly, there was no obvious relationship between either category of pDNA-PKcs peak and the presence of PRC1 proteins.

Our previous work suggested that in primary HFs, multiple PRC1 complexes co-localize at around 1000 sites in the genome [53]. Although we have no information about how these binding sites are physically distributed in the nucleus, the speckled appearance of BMI1 immunofluorescence would be consistent with such numbers and also with early reports of PRC1 staining in HFs [45], [46]. Given the density of the speckles, it was not possible to draw confident conclusions regarding the co-localization of BMI1 and γH2AX foci. Even in the U2OS cell background, where we could readily visualize representative PRC1 proteins in nuclear bodies and DDR proteins in characteristic foci, the two patterns were distinct and statistical analyses did not support the proposition that the PRC1 and DDR proteins are bound at common sites. Similar conclusions applied to ChIP-based analyses, whether at selected *Asi*SI sites that have been previously characterized or at sites identified here by genome-wide ChIP-seq. We did not observe the appearance of new PRC1 peaks or the disappearance of known peaks, following the activation of *Asi*SI, effectively ruling out the suggestion that extensive DNA damage might alter the expression of PcG target genes by displacing PRC1 complexes [35].

As our findings appeared to be at odds with the existing literature, we considered a number of possible reasons for the conflicting data. One might be the nature of the DSB and how it is repaired. *Asi*SI-induced breaks can occur at precisely the same positions on both alleles, a situation that is very unlikely to arise during radiation-induced DNA damage. The continued expression of *Asi*SI also means that the damage is persistent rather than transient and failure to repair the break could affect which proteins are recruited and over what timescale. DDR proteins are recruited to radiation-induced DSBs within minutes and a similar scenario has been proposed for PRC1 proteins, with some studies suggesting that PRC1 proteins are among the first at the scene [35], [36], [39], [41], [62], [63]. They also appear to persist for several hours although there are mixed messages in the literature about the resident times of, for example, BMI1 and MEL18 [35], [41]. We chose to sample the *Asi*SI:ER-expressing cells at the 4 h time point, when the DDR seemed to have reached a plateau, but did not find any evidence supporting the recruitment of PRC1 to the DSBs.

As we were dealing with populations of HFs, it is conceivable that *Asi*SI:ER might cut at a different subset of sites in different cells, potentially diluting our ability to detect DSBs. Although unlikely, the methylation status of *Asi*SI sites could vary from cell to cell. Alternatively, errors introduced during NHEJ could randomly destroy the recognition sites for the enzyme. However, these issues did not prevent us from detecting the DDR proteins at *Asi*SI sites in the non-clonal HF populations, either by immunofluorescence or by ChIP.

As PRC1 complexes are generally viewed as transcriptional repressors, it could be argued that they are less likely to be found on transcriptionally active genes. This could lead to an inverse correlation between PRC1 binding and the *Asi*SI sites that are sensitive to cleavage. However, there were a number of loci at which we detected OHT-dependent recruitment of pDNA-PKcs at *Asi*SI sites within PRC1-occupied regions of chromatin (e.g. *ABCC9*, *SLC32A1*, *NR6A1* etc). We did not observe the converse situation, namely the appearance of novel PRC1 peaks whose association with *Asi*SI sites is OHT-dependent.

We have suggested that the binding profiles of PRC1 components in HFs are best explained by the concept of Polycomb bodies in which multiple variants of the canonical PRC1 complex act collectively rather than individually [53]. It is conceivable, therefore, that our ability to map the PRC1 components by ChIP depends on their co-operative association with chromatin, a situation that might not apply at DSBs. While we cannot exclude this possibility, we were able to ChIP a number of DDR proteins at *Asi*SI-induced DSBs, even though the enrichment was often an order of magnitude lower than observed for PRC1 proteins at PRC1 target genes. In the few published studies that have reported binding of PcG proteins at a specific DSB, the enrichment was weak and some of the PRC1 components appeared to bind more avidly to the engineered DSBs than to bona fide PcG targets [41], [63]. We think it unlikely that the robust peaks of MEL18 that we observe at multiple sites throughout the genome somehow mask more subtle recruitment at a minority of *Asi*SI sites. In this context, we note that several of the published studies have described partial co-localization, involving only a subset of PcG and a subset of DDR proteins. Taken together, the data suggest that the proposed link between PcG proteins and DNA repair is more tenuous than presently assumed.

## Supporting Information

Figure S1 Visualizing *Asi*SI-induced DNA damage foci in U2OS cells by indirect immunofluorescence. **A**). *Asi*SI:ER-transduced U2OS cells were treated for 4 h with or without OHT and co-stained with DAPI and antibodies against either the HA-tag on the fusion protein (green) or γH2AX (red). Right panels show quantification of the HA intensity (voxels) and number of γH2AX foci in 20 representative nuclei, +/− OHT. Error bars represent the standard deviation and *** signifies a P value<0.001 in a student’s t-test. **B**. Similar analyses comparing the staining for γH2AX (red) and 53BP1, BMI1 or RING2 (green). Images were deconvoluted using Huygens Essential software and the Imaris program was used to generate a co-localization channel (yellow). Middle panel shows Pearson’s correlation coefficient values for the indicated pairs of markers in three representative nuclei. Right panel shows equivalent analyses for RING1/RING2 and BMI1/MEL18 co-localization based on additional data not shown.
(TIFF)

Figure S2 Examples of ChIP-seq data at representative *Asi*SI sites. The panels show DNA sequence tag densities following ChIP-seq with the pDNA-PKcs and MEL18 antibodies in *Asi*SI:ER-transduced Hs68 cells, before and after addition of OHT, as indicated. Input refers to parallel analyses of the chromatin before immunoprecipitation. The H3K4me3 track refers to genome-wide enrichment of H3K4me3 in the Hs68 strain of HFs from a previously deposited dataset (GEO accession number 40740). **A**. examples of clustered *Asi*SI sites on chromosomes 19 and 14. **B** and **C**. examples at which pDNA-PKcs binding extends for a considerable distance on either side of the *Asi*SI site.
(TIFF)

Figure S3 Co-localization of MEL18 and other PRC1 components at selected target loci. The panels show DNA sequence tag densities following ChIP-seq with the pDNA-PKcs and MEL18 antibodies in *Asi*SI:ER-transduced Hs68 cells, before and after addition of OHT, as indicated. The profiles are aligned with equivalent data for CBX6, CBX7, CBX8, RING1 and RING2 in normal cells, from a previously deposited dataset (GEO accession number 40740). At the *HOXD* cluster (**A**) and *GATA6* (**B**) there are no predicted *Asi*SI sites. **C**. An example where pDNA-PKcs and PRC1 binding occurs discretely on adjacent loci. D. Two examples of pDNA-PKcs peaks that are present in both OHT-treated and untreated cells and at or near a known PRC1 target.
(TIFF)

Figure S4 **Examples of ChIP-seq data where** ***Asi*** **SI sites coincide with PRC1 peaks.** **The panels show** DNA sequence tag densities following ChIP-seq with the pDNA-PKcs and MEL18 antibodies in *Asi*SI:ER-transduced Hs68 cells, before and after addition of OHT, as indicated. Input refers to parallel analyses of the chromatin before immunoprecipitation. The H3K4me3 track refers to genome-wide enrichment of H3K4me3 in the Hs68 strain of HFs from a previously deposited dataset (GEO accession number 40740). In all three examples, the pDNA-PKcs peak is specifically observed in OHT-treated cells whereas the MEL18 signal is not affected by addition of OHT.
(TIFF)

Figure S5 Examples of pDNA-PKcs peaks that are independent of *Asi*SI:ER activation. The panels show DNA sequence tag densities following ChIP-seq with the pDNA-PKcs and MEL18 antibodies in *Asi*SI:ER-transduced Hs68 cells, before and after addition of OHT, as indicated. Input refers to parallel analyses of the chromatin before immunoprecipitation. The H3K4me3 track refers to genome-wide enrichment of H3K4me3 in the Hs68 strain of HFs from a previously deposited dataset (GEO accession number 40740).
(TIFF)

Table S1 Antibodies used in this study and their applications.
(DOCX)

Table S2 Oligonucleotide primers used for analyses of ChIP DNA by qPCR. Genome co-ordinates are based on the Feb 2009, GRCh37/hg19 release of the human genome sequence according to the UCSC genome browser. Primers used in Figures 3, 6 and 7 are identified by shading.
(XLSX)

Table S3 List of *Asi*SI sites at which a pDNA-PKcs peak was detected in OHT-treated cells.
(XLS)
