# Supplementary figures and images for: Role of Polycomb Group Proteins in the DNA Damage Response – A Reassessment
Source: PLoS One. 2014 Jul 24;9(7):e102968. doi: 10.1371/journal.pone.0102968 (PMC4109945; doi:10.1371/journal.pone.0102968)

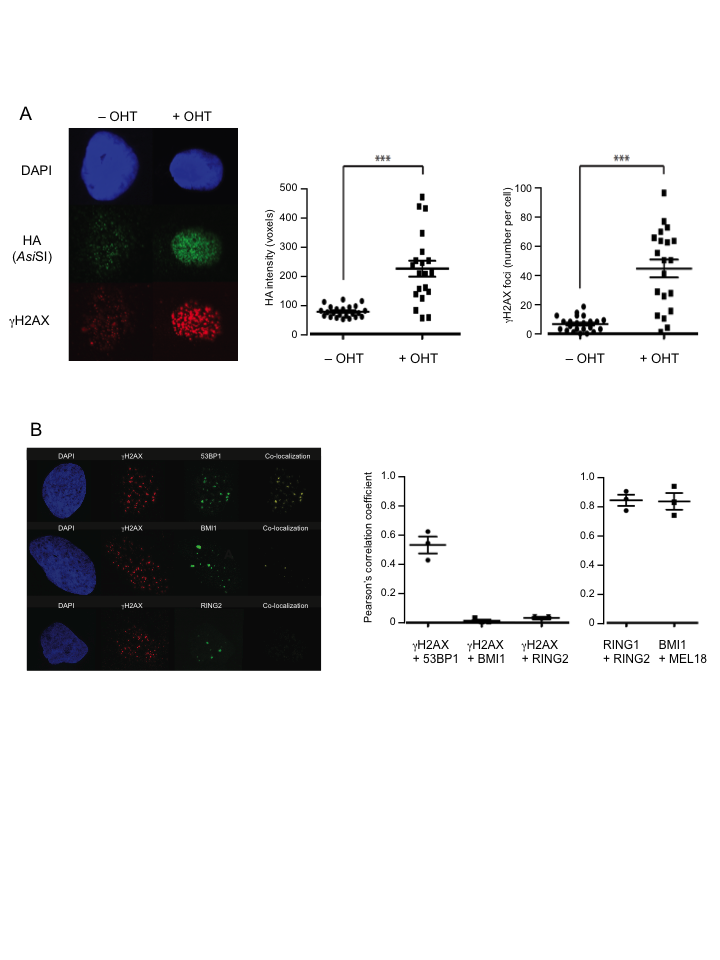

Supplement: Figure S1 — Visualizing AsiSI-induced DNA damage foci in U2OS cells by indirect immunofluorescence. A). AsiSI:ER-transduced U2OS cells were treated for 4 h with or without OHT and co-stained with DAPI and antibodies against either the HA-tag on the fusion protein (green) or γH2AX (red). Right panels show quantification of the HA intensity (voxels) and number of γH2AX foci in 20 representative nuclei, +/− OHT. Error bars represent the standard deviation and *** signifies a P value<0.001 in a student’s t-test. B. Similar analyses comparing the staining for γH2AX (red) and 53BP1, BMI1 or RING2 (green). Images were deconvoluted using Huygens Essential software and the Imaris program was used to generate a co-localization channel (yellow). Middle panel shows Pearson’s correlation coefficient values for the indicated pairs of markers in three representative nuclei. Right panel shows equivalent analyses for RING1/RING2 and BMI1/MEL18 co-localization based on additional data not shown. (TIFF) [file pone.0102968.s001.tiff]

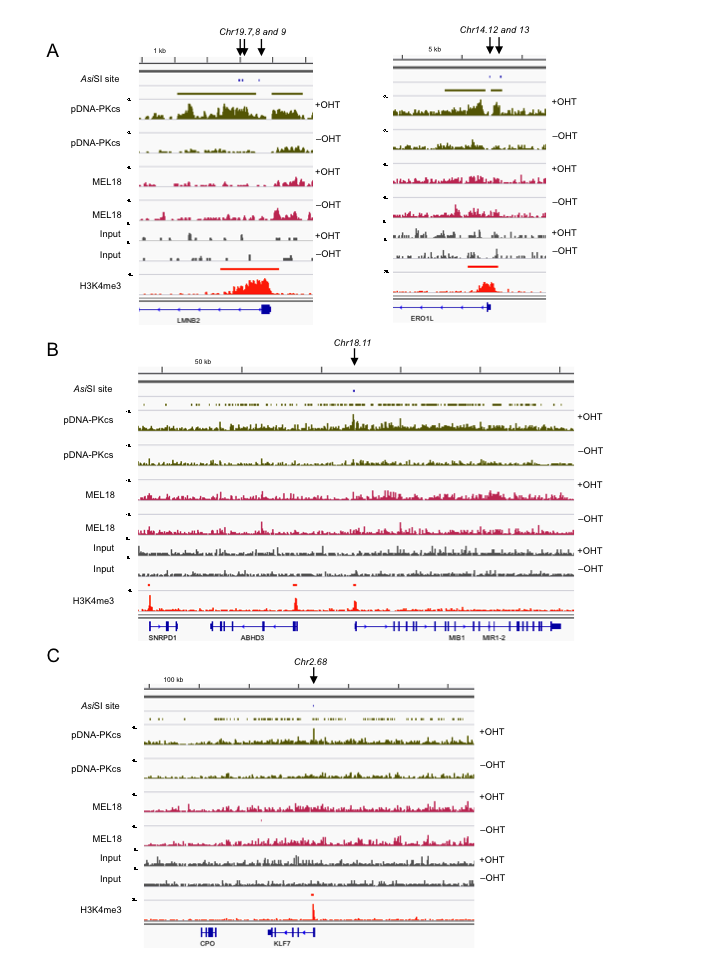

Supplement: Figure S2 — Examples of ChIP-seq data at representative AsiSI sites. The panels show DNA sequence tag densities following ChIP-seq with the pDNA-PKcs and MEL18 antibodies in AsiSI:ER-transduced Hs68 cells, before and after addition of OHT, as indicated. Input refers to parallel analyses of the chromatin before immunoprecipitation. The H3K4me3 track refers to genome-wide enrichment of H3K4me3 in the Hs68 strain of HFs from a previously deposited dataset (GEO accession number 40740). A. examples of clustered AsiSI sites on chromosomes 19 and 14. B and C. examples at which pDNA-PKcs binding extends for a considerable distance on either side of the AsiSI site. (TIFF) [file pone.0102968.s002.tiff]

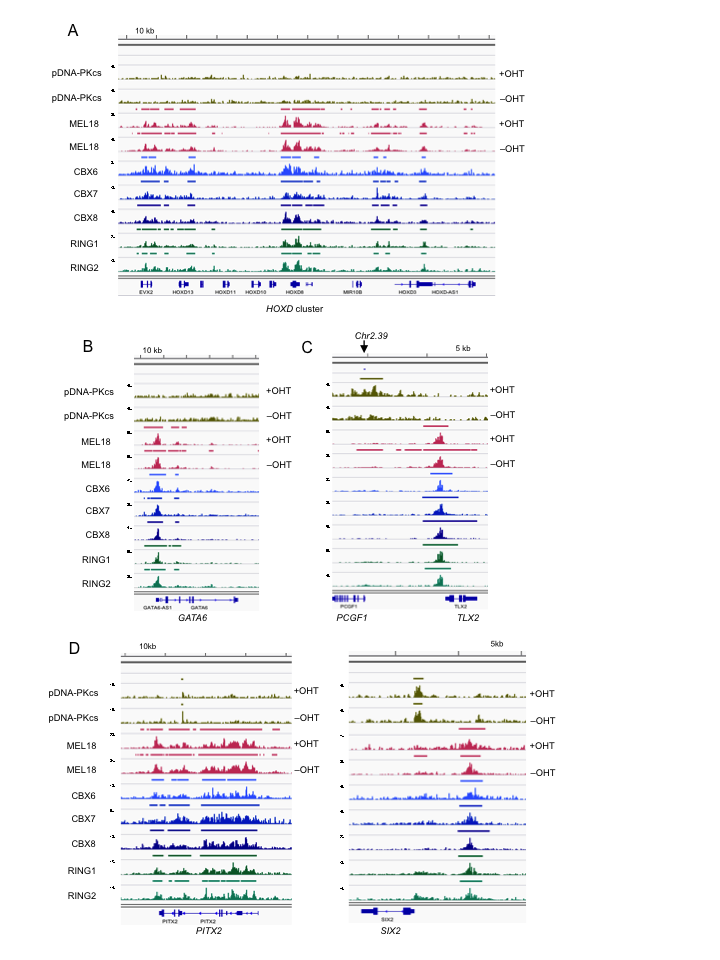

Supplement: Figure S3 — Co-localization of MEL18 and other PRC1 components at selected target loci. The panels show DNA sequence tag densities following ChIP-seq with the pDNA-PKcs and MEL18 antibodies in AsiSI:ER-transduced Hs68 cells, before and after addition of OHT, as indicated. The profiles are aligned with equivalent data for CBX6, CBX7, CBX8, RING1 and RING2 in normal cells, from a previously deposited dataset (GEO accession number 40740). At the HOXD cluster (A) and GATA6 (B) there are no predicted AsiSI sites. C. An example where pDNA-PKcs and PRC1 binding occurs discretely on adjacent loci. D. Two examples of pDNA-PKcs peaks that are present in both OHT-treated and untreated cells and at or near a known PRC1 target. (TIFF) [file pone.0102968.s003.tiff]

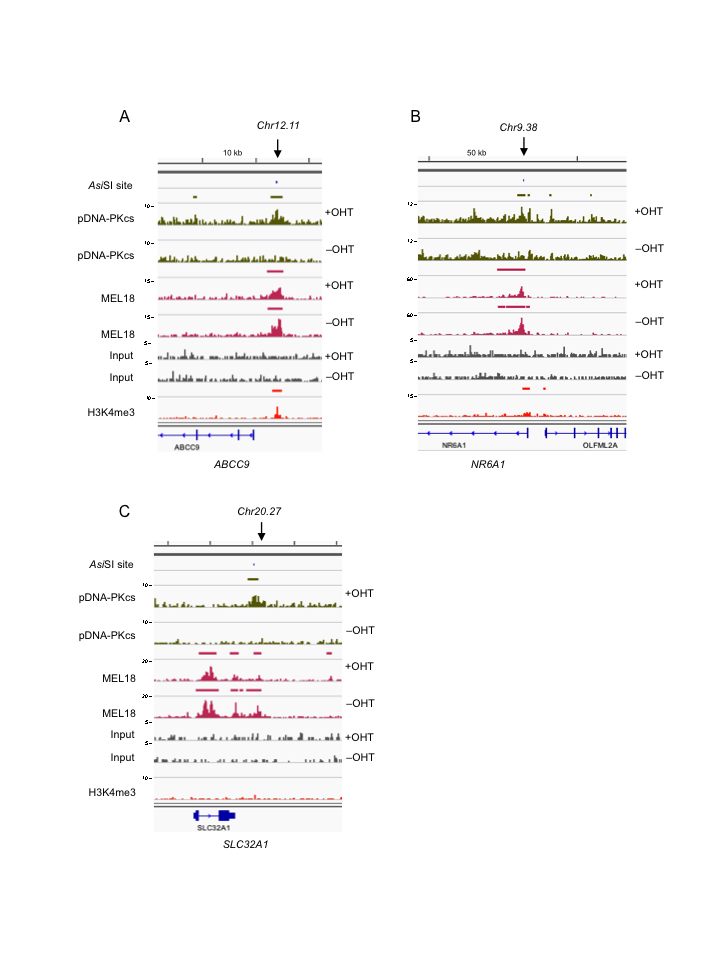

Supplement: Figure S4 — Examples of ChIP-seq data where Asi SI sites coincide with PRC1 peaks. The panels show DNA sequence tag densities following ChIP-seq with the pDNA-PKcs and MEL18 antibodies in AsiSI:ER-transduced Hs68 cells, before and after addition of OHT, as indicated. Input refers to parallel analyses of the chromatin before immunoprecipitation. The H3K4me3 track refers to genome-wide enrichment of H3K4me3 in the Hs68 strain of HFs from a previously deposited dataset (GEO accession number 40740). In all three examples, the pDNA-PKcs peak is specifically observed in OHT-treated cells whereas the MEL18 signal is not affected by addition of OHT. (TIFF) [file pone.0102968.s004.tiff]

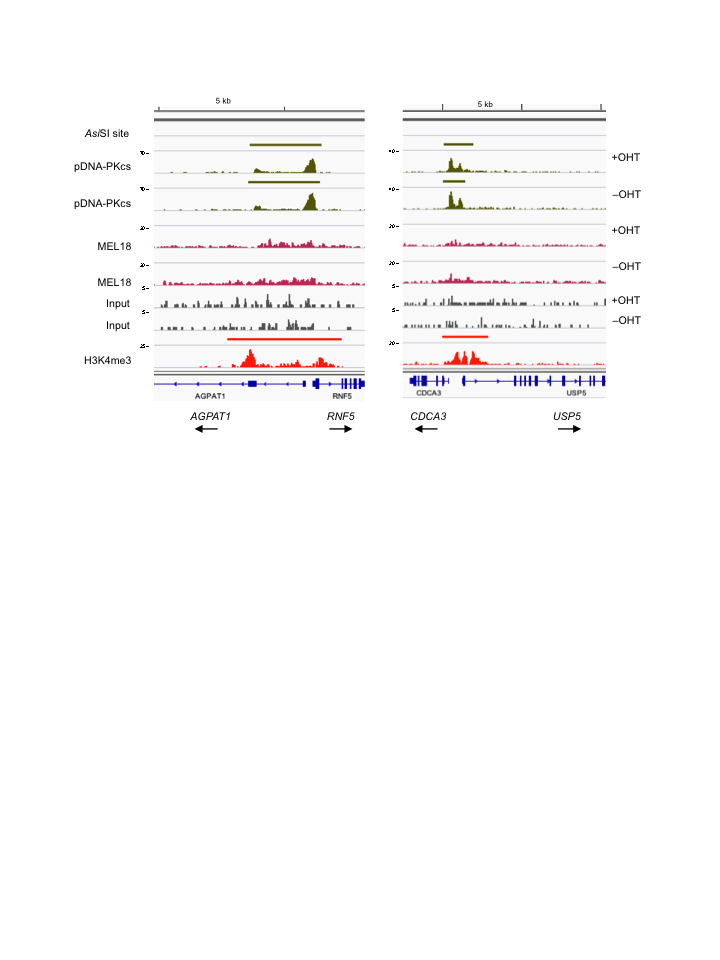

Supplement: Figure S5 — Examples of pDNA-PKcs peaks that are independent of AsiSI:ER activation. The panels show DNA sequence tag densities following ChIP-seq with the pDNA-PKcs and MEL18 antibodies in AsiSI:ER-transduced Hs68 cells, before and after addition of OHT, as indicated. Input refers to parallel analyses of the chromatin before immunoprecipitation. The H3K4me3 track refers to genome-wide enrichment of H3K4me3 in the Hs68 strain of HFs from a previously deposited dataset (GEO accession number 40740). (TIFF) [file pone.0102968.s005.tiff]
