# Supplementary material for: Role of Polycomb Group Proteins in the DNA Damage Response – A Reassessment
Source: PLoS One. 2014 Jul 24;9(7):e102968. doi: 10.1371/journal.pone.0102968 (PMC4109945; doi:10.1371/journal.pone.0102968)
Supplement: Table S1 — Antibodies used in this study and their applications. (DOCX) [file pone.0102968.s006.docx]

**Chandler et al. Supporting Information**

**Table S1 Antibodies used in this study and their applications.**

| **Target** | **Host species** | **Supplier** | **Cat. number** | **Application** |
| --- | --- | --- | --- | --- |
| HA epitope | Rabbit | Santa Cruz | sc-805 | IF |
| γH2AX(S139) | Mouse | Millipore | 05-636 | IF |
| γH2AX(S139) | Rabbit | Millipore | 07-164 | ChIP |
| 53BP1 | Rabbit | Santa Cruz | sc-22760 | IF, ChIP |
| pDNA-PKcs | Rabbit | Abcam | ab18192 | IF, ChIP |
| pATM(S1981) | Mouse | Cell Signalling | 4526 | ChIP |
| XRCC4 | Rabbit | Abcam | ab145 | ChIP |
| BMI1 | Mouse | Millipore | 05-637 | IF |
| BMI1 | Rabbit | Cell Signalling | 6694 | IF |
| BMI1 | Rabbit | Bethyl | A301-694A | ChIP |
| MEL18 | Rabbit | Santa Cruz | sc-10744 | IF, ChIP |
| RING1 | Rabbit | Cell Signalling | 2820 | IF, ChIP |
| RING2 | Mouse | Koseki lab | MAb3-3 | IF, ChIP |
| CBX6 | Rabbit | Peters lab | MRO19 | ChIP |
| H3K4me3 | Mouse | Abcam | ab1012-100 | ChIP |
| Control IgG | Rabbit | Abcam | ab46540 | ChIP |
| Control IgG | Mouse | Abcam | ab18413 | ChIP |
